# Advances in UDP-Glycosyltransferases from Medicinal Plants: Discovery, Catalytic Mechanism, Engineering and Biosynthetic Application

**DOI:** 10.3390/metabo16060402

**Published:** 2026-06-10

**Authors:** Bin Li, Qingqing Yao, Chen Li, Jiahui Li, Qiuyan Xiang, Zhiye Wang, Weiwen Lu

**Affiliations:** 1 Gansu Provincial Key Laboratory of Microbial Resources Utilization and Development, Institute of Biology, Gansu Academy of Sciences, Lanzhou 730000, China; libin2753283857@163.com (B.L.);; 2 College of Agronomy and Biotechnology, China Agricultural University, No 2 Yuanmingyuan West Road, Haidian District, Beijing 100193, China

**Keywords:** biomanufacturing, enzyme engineering, glycosylation, medicinal plants, metabolic engineering, natural products, synthetic biology, UDP-glycosyltransferases

## Abstract

Glycosylation is a critical structural modification that shapes the pharmacological properties of bioactive ingredients from Traditional Chinese Medicine (TCM), and UDP-glycosyltransferases (UGTs) are the core rate-limiting biocatalysts mediating this process. Traditional plant extraction methods are constrained by resource scarcity, long growth cycles, low target content and high environmental costs, which cannot meet the large-scale industrial demand for high-value medicinal glycosides. This review systematically outlines the latest global advances in medicinal plant UGT research, covering family classification and physiological functions, multi-omics and AI-assisted gene mining, molecular basis of substrate recognition and catalytic specificity, protein engineering for performance optimization, and the construction of full-spectrum biomanufacturing systems including in vitro multi-enzyme cascades, microbial cell factories and plant suspension cell cultures. We further discuss the core challenges of industrial scale-up, regulatory compliance and clinical translation, as well as the significant economic and technical advantages of synthetic biology-based UGT biomanufacturing platforms. This work provides a complete technical framework for the engineering application of medicinal plant UGTs, to support the green and scalable production of rare natural therapeutic glycosides.

## 1. Introduction

Natural products are an essential source for discovering new drugs. Statistics show that over the nearly four decades from 1981 to 2019, approximately half of the newly approved drugs worldwide originated directly or indirectly from natural products and their derivatives [1]. These structurally diverse and functionally unique bioactive components provide new solutions and therapeutic agents for many medical challenges, including cardiovascular diseases [2], cancer [3], osteoporosis [4], neurodegenerative diseases [5], and drug-resistant infections [6]. Compared to traditional chemically synthesized drugs, natural products often exert their therapeutic effects through multi-target and multi-pathway synergistic mechanisms, demonstrating unique clinical advantages [7]. As a treasure trove of natural products, Traditional Chinese Medicine contains a wealth of secondary metabolites with significant pharmacological activities. With breakthroughs in separation, extraction, and synthesis technologies, a series of key natural products, such as anti-tumor ginsenosides from *Panax ginseng* C.A.Mey. [8], anti-inflammatory quercetin [9], anti-malarial artemisinin from *Artemisia annua* L. [10], and anti-cancer vincristine from *Catharanthus roseus* (L.) G.Don [11], have successfully entered clinical application. However, many natural active molecules face issues such as poor water solubility, low stability, or insufficient bioavailability. Glycosylation modification is one of the effective ways to address these problems.

UGTs are key modification enzymes in the biosynthetic pathways of plant secondary metabolites, responsible for catalyzing the transfer of activated sugar donors to specific acceptor molecules, forming glycoside compounds with significant biological activities [12]. The introduction of glycosyl groups is not only the material basis for the efficacy of many TCMs, but it also significantly improves the physicochemical properties, such as water solubility and stability, as well as the pharmacokinetic characteristics of the parent compounds [13].

However, the lack of systematically characterized UGT biocatalysts, unclear catalytic mechanisms, and low efficiency of heterologous biosynthesis systems have become the core bottlenecks restricting the industrialization of TCM glycoside green manufacturing. In recent years, with the rapid development of genomics and synthetic biology technologies, significant progress has been made in the research of TCM-derived UGTs. Studies have shown that UGTs are widely involved in the biosynthesis of active TCM components, and the resulting products can be mainly divided into three categories. The first category is phenolic glycosides, such as gastrodin from *Gastrodia elata* Blume [14], salidroside from *Rhodiola rosea* L. [15], and icariside D2 from *Epimedium brevicornu* Maxim. [16]. The second category is flavonoid glycosides, such as hyperoside from *Hypericum perforatum* L. [17], kaempferol-3-rutinoside [18]. The third category is terpenoid glycosides, such as ginsenosides [19], siamenoside I from *Siraitia grosvenorii* (Swingle) C.Jeffrey ex A.M.Lu & Z.Y.Zhang [20], triterpenoid saponins [21], and carotenoid derivatives like crocins from *Crocus sativus* L. [22].

Although TCM glycosides possess immense medicinal value, traditional plant extraction methods are constrained by resource scarcity, long growth cycles, low content, and complex extraction processes, making it difficult to meet the demands of large-scale production [15]. Therefore, reducing reliance on plant resources and utilizing synthetic biology strategies to achieve the green biomanufacturing of active TCM ingredients has become an important direction for TCM modernization. In this process, UGTs serve as core catalytic components, and the mining of their gene resources, the elucidation of their catalytic mechanisms, and their engineering modifications are critical for achieving highly efficient biosynthesis [22]. Despite their profound pharmacological potential, exploiting native botanical transferases faces severe biophysical and ecological constraints. Relying exclusively upon agricultural extraction remains ecologically devastating and economically unviable due to minute accumulation levels and protracted cultivation cycles. Conversely, capturing and deploying these elusive biocatalysts is frequently paralyzed by their vast genetic redundancy, intrinsic catalytic inefficiencies, and profound functional instability when transplanted into foreign microbial environments. Overcoming this multidimensional dilemma demands a radical paradigm shift from traditional pharmacognosy toward advanced synthetic biology, necessitating a comprehensive elucidation of transferase mechanisms to enable precise structural remodeling. This article systematically reviews multi-omics mining, structural and functional analysis, protein engineering modification of UGTs, and the construction of microbial cell factories, with the aim of providing a theoretical basis for the green manufacturing of complex natural products.

## 2. Basic Characteristics and Physiological Functions of Plant UGTs

As the principal catalytic architects of plant glycosylation, UGTs constitute an ancient, expansive multigene superfamily that orchestrates the entirety of plant secondary metabolism, developmental regulation, and environmental adaptation. A systematic mastery of their structural signatures and endogenous physiological imperatives is the foundational prerequisite for the targeted mining, engineering, and synthetic application of medicinal plant UGTs.

### 2.1. Family Classification and Conserved Structural Architecture

According to the Carbohydrate-Active enZYmes (CAZy) database, plant UGTs are almost exclusively assigned to the GT1 glycosyltransferase family [23]. The sheer scale of this gene family exhibits profound species specificity, driven primarily by tandem gene duplication events throughout evolutionary history [24,25]. As of current genomic databanks, over 20,000 UGT genes have been identified across more than 100 plant species. This genetic expansion ranges from 84 in *Boehmeria nivea* (L.) Gaudich. [26], 87 in *Citrus sinensis* (L.) Osbeck [24], 91 in *Eucommia ulmoides* Oliv. [25], and 155 in *Prunus mume* (Sieb.) Sieb. et Zucc. [27], to staggering numbers exceeding 240 in specialized medicinal plants like *Panax ginseng* [28], directly mirroring the vast diversity of their respective secondary metabolomes.

The molecular foundation for this functional diversity lies in a unique structural dichotomy. The C-terminus harbors the Plant Secondary Product Glycosyltransferase (PSPG) motif, a highly conserved 44-amino acid sequence that strictly dictates the specific binding and recognition of the uridine diphosphate (UDP) moiety within activated sugar donors, with its core residues remaining essentially invariant across all plant lineages [23,29]. Conversely, the hypervariable N-terminal region forms a highly adaptable substrate-binding pocket, establishing strict recognition specificity for a myriad of aglycone acceptors. This “conserved C-terminal for sugar donors combined with a hypervariable N-terminal for aglycones” architecture provides the ultimate blueprint for their subsequent precision protein engineering [23].

### 2.2. Core Physiological Functions in Plant Tissues

Within plant metabolic networks, UGTs function as ubiquitous regulatory nodes, seamlessly integrating secondary metabolite biosynthesis, hormonal homeostasis, and stress adaptation into a unified physiological response.

The most prominent role of UGTs—and the cornerstone of their synthetic biology applications—is the terminal structural modification of secondary metabolites. By catalyzing glycosylation, UGTs drastically enhance the water solubility, structural stability, and precise tissue localization of hydrophobic aglycones [23]. This mechanism operates flawlessly across diverse chemical scaffolds. In the realm of flavonoids, specific UGTs facilitate the 3-O-glycosylation of quercetin and kaempferol in *Solanum lycopersicum* L. (SlUGT75C1) to yield antioxidant flavonol glycosides [30], drive the multi-site glycosylation of tea polyphenols in *Camellia sinensis* (L.) O.Kuntze (CsUGT72AM1) [31], and catalyze 1,2-rhamnosylation to produce bitter neohesperidosides in *Citrus sinensis* [24]. Similarly, terpenoid biosynthesis relies heavily on UGT activity, spanning the C19-O-glycosylation of ent-kaurene diterpenes to form andrographolide in *Andrographis paniculata* (Burm.f.) Nees (ApUGT12) [32], the synthesis of the safranal precursor picrocrocin in *Crocus sativus* (UGT709G1) [33], the synergistic, stepwise assembly of rare ginsenosides in *Panax ginseng* [28], and the early glycosylation of phenolic salicin in *Salix* L. [34]. UGTs are equally critical for other active compounds, such as converting emodin into active anthraquinone glycosides in *Rheum palmatum* L. [35].

This biochemical transformation inherently facilitates crucial cellular defense and compartmentalization strategies. Glycosylation enables hydrophobic toxins, active metabolites, and environmental pollutants to be safely shuttled via specific transporters into vacuoles or extracellular spaces, acting as a phase II detoxification mechanism that neutralizes potential cytotoxic damage to cytoplasmic structures [36]. In a broader ecological context, this serves as a dynamic defense reservoir. Plants routinely stockpile toxic phytoalexins as inert glycosides via UGTs, which are rapidly hydrolyzed to unleash potent chemical defenses upon pathogen invasion or herbivore attacks [29]. Interestingly, herbivorous insects like *Bemisia tabaci* (Gennadius) [37] and *Bombyx mori* L. [38] have counter-adapted through an evolutionary arms race, overexpressing their own UGTs to metabolize these very plant defenses and insecticides. Beyond biotic threats, plant UGT promoters are densely populated with cis-acting elements responsive to abiotic stressors like light, UV radiation, and heavy metals. For instance, UV-B exposure upregulates tomato *SlUGT75C1* to accumulate protective flavonoids [30], while cadmium stress triggers targeted UGT expression in *Boehmeria nivea* for heavy metal detoxification [26].

Simultaneously, UGTs operate as master regulators of plant growth and development by governing phytohormone homeostasis. Through the reversible glycosylation of signaling molecules, UGTs maintain a delicate equilibrium between active free hormones and their inactive, stored glycoside counterparts [39]. This hormonal modulation directs fundamental developmental processes, ranging from *Camellia sinensis* branching architectures governed by CsUGT74Y1-mediated auxin regulation [40] to the intricate orchestration of floral development and aromatic volatile emission in *Prunus mume* [27].

### 2.3. Functional Differentiation Across Biological Kingdoms

Despite sharing the ubiquitous PSPG motif and belonging to the same ancient GT1 superfamily, UGTs across the biological kingdom exhibit fundamental divergences in evolutionary mandate and functional positioning [29,39]. Human UGTs, predominantly localized in hepatic and intestinal tissues, are fundamentally phase II detoxification enzymes whose primary directive is to tag endogenous metabolites and exogenous pharmaceuticals for systemic excretion [36]. Similarly, insect UGTs have evolved primarily as xenobiotic resistance mechanisms to dismantle plant-derived toxins [37,38].

In stark contrast, plant UGTs are inherently designed as highly selective, core biosynthetic engines. Rather than functioning merely as waste-disposal mechanisms, they have evolved to deliberately architect complex, biologically active secondary metabolites while intricately governing endogenous growth cycles and stress responses. This strict functional dichotomy firmly delineates the scope of modern bioengineering efforts, establishing that plant-derived UGTs offer an unparalleled, highly evolved toolkit for natural product assembly that is entirely distinct from mammalian pharmacokinetic pathways. Ultimately, the systematic bioprospecting, mechanistic deciphering, and structural engineering of these medicinal plant UGTs serve as the non-negotiable prerequisites for their successful deployment in advanced synthetic biology biomanufacturing.

In summary, plant UGTs belonging to the GT1 family possess a unique “conserved C-terminal PSPG motif + hypervariable N-terminal pocket” structural architecture, and play core roles in secondary metabolite biosynthesis, stress defense and hormone homeostasis in plants. The functional differentiation between plant, human and insect UGTs also determines the unique value of plant-derived UGTs in natural product biomanufacturing.

## 3. Multi-Omics Mining and Functional Characterization of TCM UGTs

**Figure 1:**
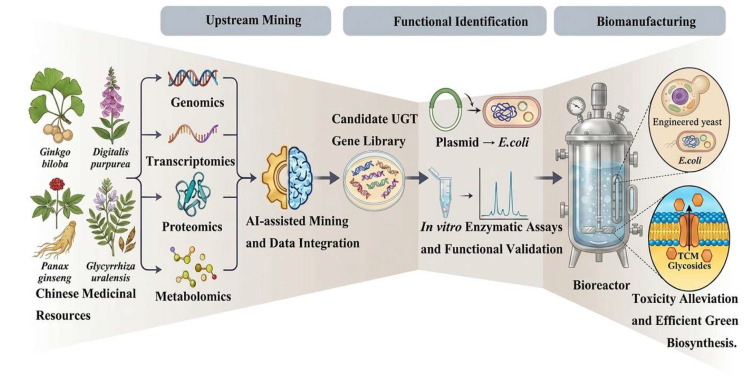
Systematic pipeline for the discovery, functional characterization, and biomanufacturing of UDP-glycosyltransferases (*UGTs*) from Traditional Chinese Medicine (TCM).

Currently, functionally validated UGTs from medicinal plants can be systematically categorized into three major functional clades corresponding to their catalytic product types. Phenolic glycoside-specific UGTs are predominantly identified from *Orchidaceae* (*Gastrodia elata*), *Apocynaceae* (*Rauvolfia serpentina* (L.) Benth. ex Kurz), and *Asteraceae* (*Artemisia annua*). Flavonoid glycoside-related UGTs are mainly sourced from *Ranunculaceae* (*Trollius chinensis* Bunge), *Fabaceae* (*Glycyrrhiza uralensis* Fisch. ex DC.), and *Berberidaceae* (*Epimedium pubescens* Maxim.). Terpenoid glycoside biosynthetic UGTs are primarily derived from *Araliaceae* (*Panax ginseng*), *Lamiaceae* (*Salvia miltiorrhiza*), and *Cucurbitaceae* (*Siraitia grosvenorii*). Targeted mining of UGT genes from the above medicinal plant clades has established a standardized technical pipeline based on multi-omics joint analysis. The rapid development of high-throughput sequencing technologies and the significant reduction in sequencing costs have propelled medicinal plant genomics and transcriptomics research into the mainstream. Compared to the less efficient early homologous cloning methods, utilizing multi-omics technologies to deeply mine key UGT genes in the biosynthetic pathways of active TCM components has become the primary strategy for deciphering the mechanisms underlying the quality formation of authentic medicinal materials (Figure 1) [41].

### 3.1. Gene Mining Based on Multi-Omics Association Analysis

Single-omics approaches often struggle to fully reveal the complexity and functional specificity of the UGT gene family. Current research generally adopts integration strategies, effectively targeting key candidate UGTs through the association analysis of transcriptomic, proteomic, and metabolomic data (Table 1). In medicinal plants, UGTs related to the synthesis of target glycoside compounds can be precisely located by comparing the metabolite accumulation profiles and gene expression profiles under different ecotypes, tissues, or treatment conditions. For example, in the study of *Artemisia annua*, by comparing the metabolic differences between low-artemisinin and high-artemisinin ecotypes, combined with transcriptomic and proteomic analyses, researchers screened 28 candidate genes from 177 annotated AaUGTs and ultimately identified the key enzyme AaUGT256 [42]. Similarly, in *Lycium barbarum* L., by comparing metabolomic and transcriptomic data across different varieties, researchers found that the expression of genes in the ascorbic acid (AA) synthesis pathway did not completely align with the content of 2-O-β-D-glucopyranosyl-L-ascorbic acid, leading to the hypothesis that the terminal glycosylation step was crucial; they subsequently targeted 10 candidate genes through phylogenetic and co-expression analyses [43].

### 3.2. Application of Targeted Proteomics

Changes at the transcriptional level do not always fully reflect the levels of functional proteins. Targeted proteomics technology can directly identify and quantify the abundance of UGT enzymes, serving as a vital supplement for functional verification. For instance, researchers identified various P450 and UGT enzymes in the brain microsomes of humans and model animals through non-targeted and targeted proteomics, providing direct evidence for understanding localized drug metabolism in the brain [50]. In the field of drug metabolism, mass spectrometry-based targeted proteomics has become the standard method for quantifying the absolute abundance of key UGT isozymes in tissues [51].

### 3.3. AI-Assisted Mining

**Figure 2:**
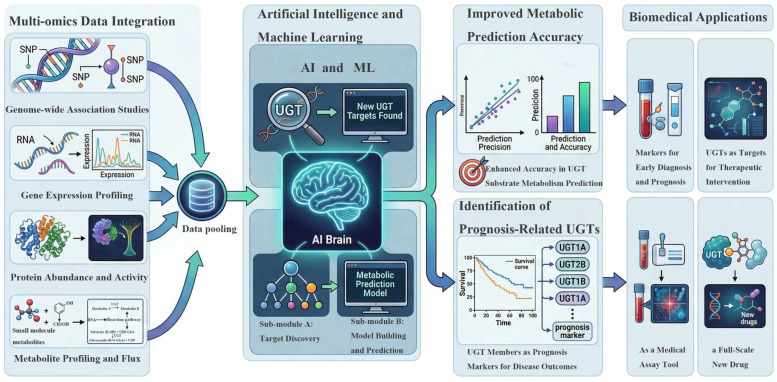
Workflow of AI-driven mining of novel *UGTs* and their applications.

With the advancement of bioinformatics, utilizing artificial intelligence (AI) algorithms to mine novel UGT-related targets and construct metabolic prediction models has become a frontier direction in this field (Figure 2) [52]. Current studies integrate multi-omics data with machine learning methods to not only improve the accuracy of predicting UGT substrate metabolism but also successfully identify UGT family members associated with disease prognosis, providing powerful tools for the discovery of novel biomarkers and the development of drug targets [53]. Furthermore, the advent of cutting-edge protein language models and advanced deep learning structural prediction tools has revolutionized the mining process. These AI-driven models enable highly accurate prediction of UGT 3D structures and dynamic substrate-binding pockets directly from sequence data, significantly accelerating the discovery of novel UGTs and bypassing time-consuming traditional crystallographic methods [52].

### 3.4. Criteria for Functional Characterization

After mining candidate UGT genes, it is necessary to clarify their catalytic properties through experiments. In vitro recombinant expression and enzyme activity analysis are the primary means of functional characterization. Typically, candidate genes are heterologously expressed in prokaryotic or eukaryotic systems and the proteins are purified; subsequently, using UDP-glucose as the sugar donor, in vitro enzymatic reactions are performed with aglycone substrates, and the generation of products is detected using HPLC-MS to confirm their catalytic functions [54]. The key UGTs that have been functionally characterized from various TCM sources, along with their specific catalytic functions, are summarized in Table 2.

In summary, multi-omics joint analysis integrating transcriptomics, proteomics and metabolomics has replaced traditional homologous cloning as the mainstream strategy for efficient mining of medicinal plant UGTs. The emerging AI-assisted mining and structure prediction methods further accelerate the screening process of candidate enzymes. Standardized in vitro enzymatic activity verification is the gold standard for confirming the catalytic function of candidate UGTs. The functional UGTs obtained through the above processes require further analysis of their catalytic mechanisms to guide subsequent engineering optimization.

## 4. Catalytic Mechanisms and Functional Characteristics of TCM UGTs

### 4.1. Substrate Selectivity and Enzyme Promiscuity

Plant UGTs exhibit a wide spectrum of catalytic characteristics, spanning from broad substrate promiscuity to highly stringent regioselectivity. Some UGTs possess a broad substrate profile; for instance, HhUGT74AG11 from *Hedera helix* L. can not only catalyze the C-28 glycosylation of oleanane-type triterpenes but also the C-7 glycosylation of flavonoids [44]. GT1 from *Cyclocarya paliurus* (Batal.) Iljinsk. even possesses O-, N-, and S-glycosylation activities for diverse backbones [63]. Meanwhile, many TCM UGTs exhibit strict regioselectivity. For example, ApUGT12 from *Andrographis paniculata* specifically catalyzes the C19-OH glycosylation of the diterpenoid andrograpanin, demonstrating strict backbone and site selectivity [62]. A common and critical misconception is that substrate promiscuity and regioselectivity are mutually exclusive. In standard enzymology, enzyme promiscuity refers to the ability of an enzyme to catalyze reactions with substrates other than its native physiological target, encompassing three main classes: substrate promiscuity, catalytic promiscuity, and regioselective promiscuity. For synthetic biology applications, the most desirable trait is substrate promiscuity paired with strict regioselectivity: this combination allows a single enzyme to process multiple structurally diverse aglycones, while producing only a single, homogeneous glycosylated product, eliminating the need for complex downstream isomer separation. This unique catalytic feature has become a core research focus in the biomanufacturing of natural products. It enables enzymes to recognize and catalyze non-natural substrates with massive structural differences, bypassing the constraints of natural plant evolutionary pathways [45]. For instance, the Bs-YjiC UGT sourced from *Bacillus subtilis* (Ehrenberg) Cohn exhibits significant substrate promiscuity, accepting over 30 different triterpenoid, flavonoid and phenolic aglycones; meanwhile, it maintains absolute regioselectivity for each substrate backbone. Through structural elucidation and rational pocket engineering, researchers have leveraged this dual property to achieve efficient biosynthesis of a series of homogeneous unnatural ginsenosides that cannot be obtained via plant extraction [61]. Biocatalytic substrate adaptability and spatial positioning strictness are two interconnected, synergistic biochemical traits, rather than fundamentally opposing properties. The former defines a transferase’s capacity to process varied chemical scaffolds, while the latter dictates consistent targeting of a single reactive locus within any provided molecule. Such phenomena frequently manifest synergistically within a single enzyme architecture [48]. From a conformational standpoint, molecule capture among botanical Family-I members relies heavily upon their amino-terminal topology, whilst the carboxy-terminal region remains intrinsically tailored for diphosphate-sugar tethering. The geometric contours, lipophilic microenvironment, and charge distribution of this receiving cavity collectively dictate spatial allowances toward exogenous small molecules [64]. Consequently, interrogations employing computational simulations, targeted residue substitutions, and three-dimensional mapping serve as indispensable strategies for deciphering, as well as rationally remodeling, predefined binding affinities. Exploiting such elasticity, synergistic with rational precursor modifications, enables investigators to circumvent strict biosynthetic hurdles inherent to native flora via cell-free cascades or engineered cellular chassis. This facilitates the biomanufacturing of anomalous glycosidic architectures harboring unprecedented core configurations [65]. Such innovations profoundly enrich the molecular inventory of complex biometabolites, delivering critical templates for optimizing pharmacokinetic profiles and catalyzing avant-garde pharmacological discoveries.

Ultimately, traditional botanical glycotransferase repertoires encompass a vast functional continuum, spanning from profoundly adaptable variants processing disparate xenobiotics to meticulously selective units operating exclusively upon predefined loci. This mechanistic versatility links directly back to the spatial plasticity of the molecule-docking cleft, prominently situated at the N-terminus. Functionally evaluated, whilst robust spatial exactitude ensures the generation of homogenous therapeutic agents, broad-spectrum accommodation serves as the paramount engine driving structural diversification.

### 4.2. Formation of Special Glycosidic Bonds

Beyond typical oxygen-linked sugar conjugations, specific botanical enzymes orchestrate the assembly of highly resilient carbon-carbon bridges. Within these unique architectures, saccharide units become covalently anchored straight onto the benzenoid frameworks of therapeutic phytocompounds. Such structural configurations endow the resulting derivatives with profound recalcitrance against both acidic degradation and physiological enzymatic cleavage. This kinetic durability holds paramount clinical significance, particularly concerning therapeutic flavonoids celebrated for their robust oxidative shielding and immune-modulating efficacies. From a thermodynamic perspective, driving direct nucleophilic engagement between a donor’s anomeric center and an electron-abundant aromatic core presents a formidable biophysical hurdle. Foundational investigations into cereal-derived biocatalysts have elucidated how specialized spatial clefts evolved to overcome this activation barrier [66]. Furthermore, achieving this intricate spatial chemistry frequently necessitates specific substrate pre-conditioning. Crucial mechanistic revelations indicate that certain catalysts bypass the steric hindrance of fully conjugated frameworks by selectively recognizing transient intermediates, notably 2-hydroxyflavanones, thereby executing the sugar transfer prior to subsequent dehydration events [67].

Consequently, exact molecular confinement within the reactive pocket dictates the ultimate regional specificity—determining whether modification transpires at the 6-C position, the 8-C locus, or via a successive dual-addition. Illustrating this remarkable precision, the isolated factor TcCGT1 from *Trollius chinensis* imposes an extraordinarily strict spatial requirement, restricting efficient sugar attachments almost exclusively to the C8 site of flavonoid backbones [45]. In a more structurally complex manner, the sequential bifunctional catalyst GgCGT characterized from *Glycyrrhiza glabra* L. gracefully executes a two-step, cascading carbon-conjugation reaction [58]. The sophisticated molecular orchestration of this tandem mechanism has been thoroughly clarified, revealing how specific plant lineages evolved specialized pathways for root-specific di-glycoside accumulation [68].

Ultimately, these carbon-bridging transferases constitute an elite enzymatic subdivision. Their evolutionary mastery in forging ultra-durable covalent ties not only enriches the pharmacological diversity of medicinal flora but also furnishes synthetic biology arsenals with premier genetic templates aimed at engineering degradation-resistant therapeutic molecules.

### 4.3. Sugar Donor Specificity

Equally crucial to spatial scaffold recognition is the precise mechanistic elucidation of how these transferases selectively sequester their energetic carbohydrate donors. Although uridine diphosphoglucose functions as the quintessential baseline metabolite across most flora, specialized catalytic machineries frequently bypass this default. Instead, they efficiently utilize an array of divergent precursors, incorporating D-galactosyl, L-rhamnosyl, xylopyranosyl, arabinose-derived, or glucuronate units. This metabolic flexibility transcends mere enzymatic curiosity; the exact physical nature of the appended saccharide strictly governs the aqueous solubility, physiological resilience, and ultimate clinical potency of the assembled phytocompound [69]. Structurally, this nucleoside capture mechanism is inextricably linked to a highly conserved domain, historically designated as the plant secondary metabolite glycosyltransferase (PSPG) signature [70]. Amino acid side chains situated within or flanking this consensus region establish intricate electrostatic networks and hydrogen-bonding scaffolds, locking the uridine and diphosphate components into an optimized catalytic geometry. Intriguingly, the evolutionary plasticity of these biocatalysts dictates that even minimal perturbations—such as singular residue mutations adjacent to the binding pocket—can drastically shift donor biases, enabling plants to adapt their chemical defense repertoires over millennia [71]. A compelling illustration of this strict affinity is observed in the ultimate biosynthetic step of glycyrrhizin within *Glycyrrhiza uralensis*. The responsible catalyst, designated UGT73P12, displays profound selectivity toward UDP-glucuronic acid rather than standard glucose. Crystallographic evidence confirms that strong ionic pairing between an arginine located at position 32 and the donor’s carboxylate functionality overwhelmingly drives this specialized recognition [60]. Fundamentally, donor selectivity remains a paramount determinant governing both the structural taxonomy and medicinal bioactivity of plant-derived derivatives. While the quintessential PSPG signature supplies the universal architectural foundation for nucleotide-tethering, it is the nuanced hyper-variability of peripheral active-site residues that directs specific carbohydrate selection. Mastering these binding heuristics is not merely an academic exercise in structural enzymology; it constitutes an absolute prerequisite for advanced biomanufacturing. Successfully harnessing microbial chassis demands a harmonious calibration between heterologous transferase efficiencies and endogenous nucleotide-sugar metabolic fluxes, ensuring sustainable yields of targeted therapeutics.

### 4.4. Drug Interactions and Biosynthetic Functions

Contemporary investigations into glycosyltransferases within traditional botanical medicine branch into two fundamentally divergent, yet equally critical, scientific trajectories. The initial paradigm investigates mammalian physiological networks, specifically evaluating how therapeutic botanical extracts interfere with human systemic clearance. Within this host-metabolism framework, numerous phytochemical constituents act as formidable modulators against human detoxification isoforms. To illustrate, specific isolates like bavachin exert pronounced suppressive effects against the 1A1 variant [72], whilst sauchinone effectively antagonizes the 2B7 subtype through non-competitive binding mechanics [73]. Such metabolic disruptions can drastically alter the elimination kinetics and biological half-life of concurrently prescribed pharmaceuticals. Recognizing this interference is clinically paramount, particularly regarding allopathic medications possessing highly constrained safety margins or those heavily reliant upon hepatic conjugative pathways for excretion [74].

Conversely, the secondary research domain focuses exclusively upon the endogenous botanical catalysts responsible for constructing these complex secondary metabolites. Rather than facilitating physiological detoxification, these native plant proteins operate as highly specialized biosynthetic machinery [75]. They typically execute terminal structural modifications that ultimately dictate the cellular compartmentalization, aqueous solubility, and intrinsic pharmacological potency of the final target molecule. For example, researchers pinpointed a distinct 73-subfamily factor as the primary engine driving the addition of glucuronic acid to oleanolic frameworks, essentially completing the Ro ginsenoside assembly [46]. Similarly, targeted molecular cloning from *G. uralensis* has successfully characterized distinct functional proteins that seamlessly orchestrate the production of vital bioactive flavonoids, namely liquiritin and related structural isomers [47].

Overall, the catalytic characteristics of medicinal plant UGTs present a rich functional continuum. Most UGTs exhibit a combination of broad substrate promiscuity and strict regioselectivity, which is their most valuable property for synthetic biology applications. The analysis of catalytic mechanisms including glycosidic bond formation and sugar donor specificity provides a theoretical basis for the rational engineering of UGTs to improve their catalytic performance.

## 5. Protein Engineering and Enzymatic Modification of TCM UGTs

Naturally sourced UGTs often suffer from issues such as low catalytic efficiency, poor stability, or suboptimal substrate profiles [76]. Optimizing their performance through protein engineering is a prerequisite for industrial application [77,78].

### 5.1. Directed Evolution and Regioselectivity

Bypassing the absolute necessity for high-resolution atomic crystallography, empirical sequence diversification stands as a potent paradigm for biocatalyst optimization. Generating vast genetic repositories via synthetic randomization techniques—coupled with massive phenotypic evaluations—facilitates the isolation of exceptionally proficient variants. A quintessential illustration involves the systemic modification of a specific transferase designated *UGTBL1*. Rigorous iterative adaptations yielded a hyperactive lineage exhibiting unprecedented positional exactitude toward gastrodin, ultimately enabling preparative mass-production within engineered cellular chassis [79]. Similarly transformative outcomes emerged during the artificial adaptation of the PjUGT10 protein, which governs pseudoginsenoside biogenesis. Guided mutagenesis amplified its processing capacities for both aglycones and nucleotide-sugars by astonishing factors exceeding six thousand and two thousand, respectively [78].

Refining spatial and enantiomeric discrimination constitutes a dominant trajectory within contemporary molecular engineering. Complex phytochemicals inherently possess numerous reactive endpoints, theoretically allowing for heterogeneous carbohydrate attachments. While stringent spatial precision remains mandatory for yielding homogenous pharmaceutical profiles, investigators consistently encounter a frustrating inverse correlation between positional strictness and absolute kinetic throughput [80]. Conquering this biophysical dilemma, while concurrently broadening a catalyst’s repertoire to accept economically viable or unconventional nucleotide-sugars, represents a pinnacle achievement. The recent discovery and rational restructuring of highly permissive transferases—such as specific xylosyl-conjugating enzymes—successfully unlock the biomanufacturing of unprecedented structural derivatives utilizing alternative carbohydrate donors [81].

Moving beyond entirely stochastic diversification, mathematically guided paradigms now dominate the enhancement of biocatalytic proficiency. Resolving the aforementioned enzymatic trade-offs frequently necessitates integrating wet-lab trials with advanced structural modeling. Recent paradigms demonstrate how combining empirical evaluations with sophisticated molecular simulations elegantly dictates the regional preferences of these biological machines toward highly complex polyphenolic frameworks [55]. By leveraging computational docking alongside evolutionary mapping, scientists restrict randomization exclusively to focal amino acids lining the internal reactive cavity. This targeted strategy drastically shrinks required mutational landscapes while exponentially elevating the likelihood of isolating advantageous phenotypes [82,83].

At bottom, any evolutionary campaign’s triumph relies on robust analytical pipelines. While hyphenated mass spectrometry guarantees definitive structural confirmation regarding attachment sites, primary optical or fluorometric triage systems acting to quantify nucleotide cleavage serve as indispensable preliminary filters. Synthesizing these multifaceted diagnostic layers ensures the rapid discovery of optimized machineries featuring superior kinetics and shifted molecular biases [36].

In essence, artificial molecular adaptation offers a supreme trajectory for overcoming inherent biocatalytic limitations. The remarkable transformations witnessed in previously mentioned transferases prove that continuous mutagenic iterations profoundly elevate functional performance. Upcoming breakthroughs will undoubtedly stem from merging stochastic variation with rationally guided library construction and advanced phenotypic diagnostics, perfectly equipping these biological platforms for massive therapeutic manufacturing.

### 5.2. Rational Design

Transitioning from empirical randomization, the contemporary enzymatic frontier is increasingly dominated by deterministic, architecturally guided modifications. The advent of high-resolution crystallographic methodologies, synergized with sophisticated kinetic trajectory computations, has propelled targeted protein remodeling to unprecedented heights. Notably, deep-learning algorithms have completely revolutionized this paradigm; neural network-driven conformational forecasting—exemplified by groundbreaking platforms like AlphaFold—now successfully delineates reactive cavities even where empirical X-ray diffraction data remain entirely absent [84].

**Figure 3:**
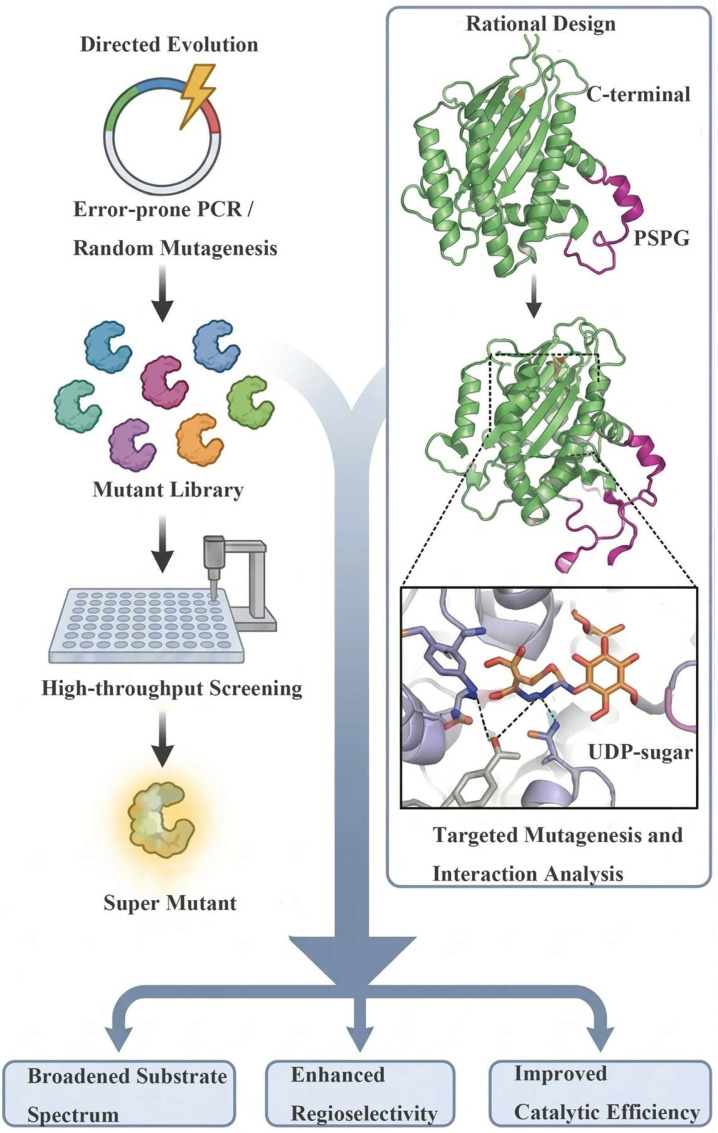
Protein engineering strategies for optimizing the catalytic performance and structural properties of plant-derived UGTs.

By strategically reconstructing these focal reactive crevices, investigators can substantially optimize intermolecular affinities, thereby elevating overall bioconversion velocities (Figure 3) [85]. Such deliberate molecular engineering relies heavily upon precisely deciphering the spatial arrangements and critical amino acid interactions uniting the exogenous precursor with the biocatalytic core [61]. Extensive bio-physical evaluations confirm that the universally conserved carboxy-terminal signature fundamentally dictates nucleotide-sugar preferences [86]. A compelling manifestation of this precision biochemistry involves the specific HtUGT72AS1 catalyst; substituting its critical tyrosine residue at position 377 with an aliphatic alanine radically amplified its processing capability toward unconventional acetylated amino-sugar donors [59].

Integrating these artificially generated topologies with advanced computational docking provides profound insights into cavity elasticity, molecule anchoring, and carbohydrate discrimination. Ultimately, manipulating the functional polarity between the aglycone-capturing amino-terminus and the nucleotide-tethering car-boxy-terminus allows scientists to overcome native botanical boundaries. This focused methodology not only shifts carbohydrate biases but dramatically enhances thermal resilience and enzymatic tolerance against harsh industrial cosolvents.

Consequently, when contrasted with stochastic methodologies, mathematically directed alterations offer a profoundly elegant and mechanistically transparent pathway. By systematically interrogating the structural determinants governing target recognition and pocket dynamics, researchers possess the ultimate blueprint for tailoring medicinal plant transferases toward massive commercial biosynthesis. Navigating the extreme structural plasticity of transferase binding pockets represents a formidable challenge that static crystallographic snapshots historically failed to resolve. Botanical UGTs frequently rely upon profound conformational flexibility—particularly within their aglycone-capturing amino-terminal domains—to accommodate massively divergent and bulky secondary metabolites. While early deep-learning architectures revolutionized unliganded protein forecasting, capturing the dynamic induced-fit mechanics of complex substrate engagements remained elusive. The recent advent of diffusion-based generative models, most notably AlphaFold 3, has fundamentally dismantled this barrier [87]. By predicting the precise joint structures of multidimensional biomolecular complexes, encompassing interacting proteins, non-canonical ligands, and varied cofactors, these avant-garde algorithms provide unprecedented spatial accuracy. When synergistically coupled with advanced molecular dynamics simulations, this computational framework elegantly delineates the multi-state thermodynamic trajectories inherent to highly flexible active sites. Consequently, structural engineers can meticulously track transient conformational shifts and mathematically redesign pocket elasticity. This predictive dynamism explicitly empowers the rational accommodation of hyper-complex, non-natural scaffolds, decisively overcoming the historical constraints of static structural enzymology.

### 5.3. Mining of Novel Enzyme Resources

Beyond the artificial remodeling of known biocatalysts, excavating inherently superior transferases directly from biological reservoirs constitutes a profoundly significant paradigm. Billions of years of relentless evolutionary pressure have already sculpted highly proficient molecular machines. A striking illustration of this innate elasticity is observed in a specific factor isolated from the herbaceous species *Helleborus thibetanus* Franch. Designated as HtUGT72AS1, this remarkable protein exhibits extraordinary functional adaptability, flawlessly processing half a dozen distinct nucleotide-linked carbohydrates through bidirectional mechanisms. Such unprecedented versatility perfectly accommodates the single-step biomanufacturing of highly complex molecular conjugates without demanding prior mutagenic interventions [59].

While eukaryotic transcriptomic pools continually unveil factors driving species-specific secondary metabolism, prokaryotic DNA libraries offer entirely distinct, yet highly complementary, biochemical advantages. Bacterial counterparts frequently display immense structural permissiveness toward xenobiotics, coupled with extremely robust heterologous expression profiles. Illustrating this cross-kingdom utility, specific agents sourced from *Bacillus subtilis* biomes—notably the Bs-YjiC and UGT109A1 proteins—have revolutionized the structural diversification of massive triterpene scaffolds. These prokaryotic machineries effectively bypass stringent native pathways, enabling the synthetic assembly of entirely artificial ginsenoside analogs featuring non-canonical linkages [61]. Furthermore, probing complex environmental microbiomes unlocks uncharted enzymatic territories that flawlessly compensate for eukaryotic limitations. Synthesizing these diverse discovery pipelines ultimately supplies the comprehensive genetic inventory required for sustainable therapeutic biomanufacturing, or at minimum, establishes robust baseline templates for subsequent directed remodeling.

Cultivating this diverse enzymatic portfolio remains the definitive strategy for navigating the complexities of synthetic biology. The existence of highly permissive factors powerfully validates that ecological adaptation has already sculpted catalysts possessing profound substrate promiscuity and immense kinetic prowess. Merging multi-level phytochemistry with deep ecological screening will undoubtedly accelerate the generation of next-generation pharmaceutical architectures, seamlessly linking natural diversity with advanced industrial biosynthesis [88].

In conclusion, protein engineering and novel enzyme resource mining provide complementary systematic solutions to overcome the inherent limitations of wild-type UGTs, including low catalytic activity, narrow substrate scope, insufficient regioselectivity, and poor operational stability. Directed evolution enables significant performance improvement without prior structural information, while structure-guided rational design supported by AI structural prediction achieves precise targeted modification based on mechanistic insights. In parallel, mining naturally efficient UGTs from plants, microorganisms, and metagenomic resources continuously expands the available biocatalytic toolbox. The integration of these three approaches will be essential for developing robust, high-performance UGT catalysts to support the scalable biomanufacturing of TCM glycosides.

## 6. Construction and Application of Biomanufacturing Systems for TCM UGTs

Combining highly efficient UGTs with synthetic biology strategies to construct in vitro multi-enzyme systems or engineered microbial cell factories is the core to achieving the green and efficient production of high-value TCM glycosides.

### 6.1. In Vitro Multi-Enzyme Cascades and Cofactor Regeneration

Liberating biotransformation processes from the unpredictable metabolic fluxes of living microorganisms grants investigators unparalleled stoichiometric governance. Constructing isolated, cell-free architectural platforms permits absolute regulation over biocatalyst ratios, microenvironmental parameters, and exogenous precursor loading. Such precisely managed environments prove particularly indispensable when handling complex therapeutic frameworks that either severely disrupt microbial viability or exhibit profound aqueous insolubility [80]. Nevertheless, the exorbitant financial penalty linked to continuous nucleotide-carbohydrate consumption historically crippled the commercial scalability of these in vitro architectures. To circumvent this prohibitive economic barrier, contemporary researchers have ingeniously engineered self-sustaining biochemical loops. By tethering the primary glycosylation event to a secondary enzymatic regeneration module—most notably leveraging sucrose cleavage proteins—scientists can continuously replenish depleted uridine diphosphate carriers utilizing exceedingly inexpensive disaccharides. This elegant thermodynamic push significantly elevates kinetic thresholds while drastically shrinking operational expenditures. A quintessential demonstration of this strategy involved integrating a specific transferase originating from *Indigofera tinctoria* L. within an autonomous recycling cascade. This optimized closed-loop network dramatically propelled gastrodin bioconversion efficiencies, ultimately capturing an astonishing ninety-three percent substrate transformation rate without demanding continuous expensive donor supplementation [57]. This self-regenerating biochemical blueprint extends seamlessly into the manufacturing of highly complex triterpenoid scaffolds. By orchestrating a rationally optimized biocatalyst alongside dynamic cofactor replenishment, investigators successfully achieved unprecedented macroscopic yields of the therapeutic Rg3 ginsenoside, registering an impressive volumetric productivity nearing ten grams per liter [89].

Essentially, constructing robust, completely decoupled synthetic assemblies effectively neutralizes the primary financial deterrents associated with cell-free synthesis. By completely bypassing the chaotic regulatory networks inherent to cellular biology, engineers can relentlessly pursue maximum stoichiometric efficiency. While optimizing overall catalyst longevity and resolving hydrophobic aglycone precipitation demand ongoing scientific scrutiny, these elegantly orchestrated recycling networks unequivocally bridge the massive chasm between fundamental mechanistic enzymology and commercially viable phytochemical mass-production [56,57].

### 6.2. Microbial Cell Factories and Metabolic Network Regulation

Harnessing genetically reprogrammed microbial populations offers an immensely scalable avenue for the mass biomanufacturing of phytochemicals. This bioindustrial paradigm typically bifurcates into two distinct operational modes: intact microbial bioconversion relying upon exogenously supplied precursors, and absolute bottom-up pathway reconstruction from fundamental carbon sources. When deploying intact cells exclusively for bioconversion, investigators prioritize maximizing enzymatic turnover while fortifying cellular resilience against xenobiotic toxicity. Demonstrating this operational efficiency, deploying the hyperactive SMWRW1W2 variant facilitated unprecedented intact-microbe phenolic bioprocessing, achieving macroscopic titers exceeding one gram per liter [79]. Parallelly, implementing a distinct transferase within a recombinant prokaryotic host secured a near-perfect ninety-eight percent transformation efficiency, yielding substantial quantities of therapeutic derivatives from simpler hydroxybenzoic precursors [56].

Conversely, executing absolute bottom-up biosynthesis demands the intricate amalgamation of upstream skeletal construction with downstream carbohydrate attachment inside a unitary chassis. Such monumental engineering endeavors necessitate the profound orchestration of nucleotide-sugar pools, redox homeostasis, and precursor routing. To forcibly redirect intracellular carbon fluxes toward critical intermediate junctions—such as enhancing malonyl-CoA accumulation—scientists frequently deploy system-wide computational modeling to pinpoint and execute minimal genomic interventions, thereby eliminating competitive biological sinks [90]. Furthermore, when the profound metabolic penalty of harboring an entire biosynthetic cascade severely compromises host viability, distributing the molecular workload across meticulously engineered microbial consortia provides an elegant strategy to alleviate intracellular stress and optimize overall productivity [91,92].

Moving beyond quintessential model hosts, unconventional lipogenic organisms are rapidly emerging as formidable biomanufacturing platforms. Leveraging the extraordinary acetyl-CoA availability and unique lipid-metabolism profile of *Yarrowia lipolytica* recently established a highly robust framework distinctly suited for assembling complex flavonoid architectures and their corresponding sugar-linked variants [93]. Naturally, the successful implementation of any synthetic cascade relies intimately upon characterizing functional botanical genes. Unlocking specific molecular determinants that govern endogenous phytochemical accumulation, such as the recent functional decoding of SlUGT75C1 from *Solanum lycopersicum*, directly furnishes synthetic biologists with highly specialized blueprints for reconstructing exact biological pathways heterologously [30].

Fundamentally, cellular factories represent the definitive trajectory for sustainable pharmaceutical procurement. Continual advancements in systems biology, coupled with the exploitation of both traditional and non-conventional host systems, will undeniably escalate volumetric yields, ensuring the economic viability of therapeutic glycoside commercialization. Reconstructing entire multi-enzyme biosynthetic cascades frequently presents insurmountable biochemical barriers for conventional prokaryotic hosts. The sheer volume of membrane-bound cytochromes and sequential specific carbohydrate additions overwhelms bacterial metabolic capacities [94]. Consequently, deploying undifferentiated botanical cell suspensions emerges as a profound biotechnological imperative for these extreme architectural assemblies. Cultured phytocells inherently possess the necessary eukaryotic endomembrane architecture and vast endogenous precursor reservoirs, facilitating the seamless mass-accumulation of highly complex medicinal molecules without requiring absolute genomic reconstruction. Integrating these higher-order cellular platforms circumvents the strict enzymatic incompatibilities plaguing standard microbial chassis [95,96].

Despite significant advances in microbial cell factories for medicinal glycoside production, microbial chassis face inherent, insurmountable physiological limitations when manufacturing highly complex, multi-modified glycosylated phytochemicals. The complete de novo biosynthetic pathways for high-value active ingredients including paclitaxel, vinca alkaloids and ginsenosides require the coordinated expression of 20–50 distinct functional enzymes, covering multiple endoplasmic reticulum-localized cytochrome P450 oxidases for backbone modification, as well as sequential site-specific glycosylation catalyzed by distinct UGT isoforms [94]. Conventional prokaryotic *Escherichia coli* (Migula) Castellani & Chalmers chassis lack the eukaryotic endomembrane system required for correct folding and functional localization of P450 enzymes. Meanwhile, heterologous reconstruction of these ultra-long, multi-gene pathways in *Saccharomyces cerevisiae* Meyen ex E.C.Hansen consistently induces severe metabolic burden, redox imbalance and pathway flux dysregulation [90].

For these structurally complex glycosides, plant suspension cell culture remains the only technically feasible and commercially scalable production platform to date. This system perfectly combines the intact native biosynthetic capacity of plant cells with the controllable operation of industrial fermentation, having achieved verified large-scale commercial success [94]. For example, optimized *Panax ginseng* suspension cell lines, combined with targeted methyl jasmonate elicitation, can achieve total ginsenoside titers above 3 g/L, and have successfully entered pilot-scale industrial production [95]. Furthermore, large-scale *Taxus chinensis* (Pilg.) Rehder cell culture has become a core commercial source of paclitaxel globally, with optimized titers exceeding 100 mg/L. Similarly, *Catharanthus roseus* suspension cell systems continue to serve as the dominant industrial platform for the production of vincristine and vinblastine bisindole alkaloids [94,95]. Notably, regardless of the chassis selected, intracellular accumulation of high-concentration target glycosides inevitably triggers cytotoxicity and limits further yield improvement, which can be effectively addressed by engineering efficient product efflux systems.

### 6.3. Product Efflux Systems and Chassis Toxicity Relief

Within bioengineered cellular hosts, the excessive internal pooling of specialized metabolites frequently induces severe cytotoxic effects, thereby imposing a formidable ceiling on commercial manufacturing capacities. Highly lipophilic phytochemicals, notably complex polyphenolics alongside triterpenoid scaffolds, are particularly notorious for penetrating and destabilizing cellular phospholipid boundaries. Such architectural perturbations catastrophically dissipate vital electrochemical gradients and paralyze central biochemical pathways [96].

To circumvent these biological limitations, investigators increasingly look toward nature’s innate detoxification machinery. Botanical systems have developed sophisticated integral membrane proteins to orchestrate the spatial compartmentalization and outward translocation of defensive compounds [97]. A paramount illustration involves the ATP-binding cassette (ABC) architecture. Recent physiological characterizations within *Salvia miltiorrhiza* Bunge demonstrate how a specialized pump forcefully extrudes diterpenoid tanshinones outward from peridermal tissues [98]. Furthermore, analogous mechanisms govern the systemic mobility of related polyphenols, as evidenced by specific pump variants isolated from *Medicago truncatula* Gaertn. orchestrating isoflavonoid localization [99]. Translocating such highly efficient botanical export pumps into synthetic microbial platforms establishes a dynamic “production-secretion” equilibrium. This active expulsion effectively mitigates localized cellular poisoning, ensuring sustained biocatalyst viability. Nevertheless, the heterologous integration of major facilitator superfamilies or pleiotropic drug resistance networks is not devoid of metabolic friction. Unregulated overexpression of these transmembrane domains frequently drains intracellular adenosine triphosphate pools and compromises structural envelope integrity. Consequently, establishing an energetically sustainable transport architecture—minimizing parasitic resource consumption while maximizing product expulsion—constitutes a critical balancing act for microbial engineers [100].

### 6.4. Challenges in Industrial Scale-Up and Clinical Translation

Compared to traditional plant extraction processes, glycosyltransferase-based biomanufacturing platforms driven by synthetic biology demonstrate significant economic and technical advantages. For instance, the production cost of salidroside via microbial fermentation has decreased by over 65% compared to traditional extraction [101], while the fermentation yield of ginsenoside compound K has increased nearly a hundredfold [102], drastically reducing production costs and alleviating the ecological pressures associated with harvesting wild medicinal plants. However, the large-scale industrialization and clinical translation of this technology still face several challenges. First, as the primary cost driver in glycoside biosynthesis, the uridine diphosphate sugar (UDP-sugar) regeneration system lacks robust stability in 50-cubic-meter industrial fermenters, causing its cost share to surge from under 15% at the laboratory scale to over 40% during industrial production [103]. Second, genetically engineered chassis strains often exhibit genetic and metabolic instability during large-scale fermentation and continuous subculturing due to plasmid loss, metabolic burden, and gene silencing, resulting in a 30% to 50% yield decline compared to shake-flask levels [104]. Furthermore, regarding regulatory compliance, agencies such as the FDA and NMPA impose stricter requirements on active pharmaceutical ingredients (APIs) derived from synthetic biology than on traditional plant-sourced materials, mandating full-process traceability data, impurity profiling, and comprehensive safety evaluations [105]. Despite these bottlenecks, breakthrough progress has been made in clinical translation; biosynthetic gastrodin and salidroside have been successfully validated for pharmacological and pharmacokinetic equivalence with their plant-derived counterparts, and biosynthetic gastrodin API has notably gained regulatory approval and achieved commercial mass production in China [106]. Moving forward, it is imperative to establish standardized quality evaluation systems that align with international regulatory guidelines to facilitate the entry of more high-value, rare Traditional Chinese medicine glycosides into preclinical studies, clinical trials, and commercialization.

To sum up, in vitro multi-enzyme cascades, engineered microbial cell factories and plant suspension cell culture systems form a complete biomanufacturing technology system for TCM glycosides, adapting to the production requirements of products with different structural complexities. The introduction of product efflux systems further breaks through the yield bottleneck caused by chassis cytotoxicity. Promoting the scale-up of these systems from laboratory to industrial scale is the core direction of future research.

## 7. Conclusions

Research targeting botanical carbohydrate-transferring enzymes has transitioned from rudimentary sequence discovery toward profound mechanistic elucidation and rational structural remodeling, ultimately establishing the foundational framework for robust biomanufacturing platforms.

Moving forward, several critical trajectories will define the future development of this discipline. First, integrating advanced multi-omics technologies with deep ecological screening will continuously unveil novel transferases possessing unprecedented regio- and stereochemical exactitude. Second, deploying artificial intelligence alongside advanced structural predictive modeling will revolutionize targeted sequence mutagenesis, replacing stochastic empirical trials with deliberate, mechanism-driven functional evolution. Third, interrogating the underlying regulatory dynamics of these transferases within native plant stress-response networks will provide vital genetic targets for the agronomical improvement of high-quality medicinal species. Fourth, constructing highly resilient cellular factories—driven by optimized multidimensional cascade assemblies and precise carbon-flux routing—will accelerate the commercial green production of high-value medicinal glycosides.

Crucially, among these distinct future avenues, conquering the engineering friction inherent to massive industrial scale-up undeniably represents the most immediate and critical bottleneck restricting the development of this entire field. Overcoming the exorbitant financial burden of nucleotide-sugar donor regeneration while simultaneously guaranteeing absolute metabolic stability within large-scale industrial bioreactors dictates the ultimate commercial viability of engineered medicinal glycosylation. Successfully navigating this profound translation from bench-scale scientific triumphs to economically competitive industrial manufacturing remains the paramount overarching challenge for next-generation synthetic biology applications in this domain.

## Figures and Tables

**Table 1:** Statistics on the Size of *UGT* Gene Families in Representative Medicinal Plants.

Medicinal Plant Species	Number of UGT Gene Family	Functionally Characterized Subfamilies	References
*Gastrodia elata*	50	*UGT7204*, *UGT7311*, *UGT7315*	[14]
*Artemisia annua*	177	*UGT71*, *UGT73*, *UGT85*	[42]
*Lycium barbarum*	130+	*UGT78*, *UGT79*, *UGT85*	[43]
*Hedera helix*	120+	*UGT74*, *UGT85*	[44]
*Trollius chinensis*	110+	*UGT71*, *UGT72*	[45]
*Panax ginseng*	240+	*UGT71*, *UGT73*, *UGT74*, *UGT94*	[46]
*Glycyrrhiza uralensis*	150+	*UGT71*, *UGT73*, *UGT79*, *UGT84*	[47]
*Epimedium pubescens*	339	*UGT78*, *UGT79*, *UGT91*	[48]
*Salvia miltiorrhiza*	140+	*UGT71*, *UGT75*, *UGT85*	[49]

**Table 2:** Summary of Core Functionally Characterized *UGTs* Sourced from Traditional Chinese Medicine.

Category	Gene Name	Source Medicinal Plant	Aglycone Substrate	Catalytic Site/Core Function	References
Phenolic Glycosides	*UGTBL1*	*Morinda officinalis*	p-Hydroxybenzyl alcohol	C-OH glycosylation, synthesizing gastrodin	[55]
	*RsUGT*	*Rauvolfia serpentina*	p-Hydroxybenzyl alcohol	Synthesizing gastrodin	[56]
	*itUGT2*	*Indigofera tinctoria*	p-Hydroxybenzyl alcohol	Synthesizing gastrodin	[57]
	*AaUGT256*	*Artemisia annua*	Phenylpropanoid aglycones	Phenylpropanoid glycoside synthesis	[42]
Flavonoid Glycosides	*TcCGT1*	*Trollius chinensis*	Flavonoid aglycones	8-C-glycosylation, synthesizing stable C-glycosides	[45]
	*GgCGT*	*Glycyrrhiza glabra*	Flavonoid aglycones	Two-step sequential C-glycosylation	[58]
	*GuUGT2/GuUGT3*	*Glycyrrhiza uralensis*	Liquiritigenin	7-O-glycosylation, synthesizing liquiritin/isoliquiritin	[47]
	*HtUGT72AS1*	*Helleborus thibetanus*	Phenolic/Flavonoid aglycones	Broad-spectrum glycosylation, reversible catalysis	[59]
Terpenoid Glycosides	*PgUGAT252645*	*Panax ginseng*	Oleanolic acid	C3-glycosylation, synthesizing ginsenoside Ro	[46]
	*HhUGT74AG11*	*Hedera helix*	Oleanane-type triterpenes	C28-glycosylation, synthesizing triterpenoid saponins	[44]
	*UGT73P12*	*Glycyrrhiza uralensis*	Glycyrrhetinic acid	C3-glycosylation, synthesizing glycyrrhizin	[60]
	*Bs-YjiC*	*Bacillus subtilis*	Ginsenoside aglycones	Broad substrate promiscuity, unnatural ginsenoside synthesis	[61]
	*ApUGT12*	*Andrographis paniculata*	Andrograpanin	C19-OH specific glycosylation	[62]

## Data Availability

Data sharing is not applicable to this article as no new data were created or analyzed in this study.

## Abbreviations

The following abbreviations are used in this manuscript:

TCM	Traditional Chinese Medicine
UGT	UDP-glycosyltransferase
AA	Ascorbic acid
CAZy	Carbohydrate-Active enZYmes
AI	Artificial intelligence
HPLC-MS	High-performance liquid chromatography-mass spectrometry
UDP-GlcA	UDP-glucuronic acid
PCR	Polymerase chain reaction
MD	Molecular dynamics
PSPG	Plant Secondary Product Glycosyltransferase
pHBA	p-Hydroxybenzyl alcohol
ABC	ATP-binding cassette
